# Recent insights and advances in gut microbiota's influence on host antiviral immunity

**DOI:** 10.3389/fmicb.2025.1536778

**Published:** 2025-02-27

**Authors:** Ying Liu, Danying Yan, Ran Chen, Yingying Zhang, Chuwen Wang, Guoqing Qian

**Affiliations:** ^1^Health Science Center, Ningbo University, Ningbo, Zhejiang, China; ^2^Department of Infectious Diseases, The First Affiliated Hospital, Ningbo University, Ningbo, Zhejiang, China; ^3^Cixi Biomedical Research Institute, Wenzhou Medical University, Wenzhou, Zhejiang, China

**Keywords:** gut microbiota, microbiome, virome, antiviral immunity, metabolites

## Abstract

A diverse array of microbial organisms colonizes the human body, collectively known as symbiotic microbial communities. Among the various pathogen infections that hosts encounter, viral infections represent one of the most significant public health challenges worldwide. The gut microbiota is considered an important biological barrier against viral infections and may serve as a promising target for adjuvant antiviral therapy. However, the potential impact of symbiotic microbiota on viral infection remains relatively understudied. In this review, we discuss the specific regulatory mechanisms of gut microbiota in antiviral immunity, highlighting recent advances in how gut microbiota regulate the host immune response, produce immune-related molecules, and enhance the host's defense against viruses. Finally, we also discuss the antiviral potential of oral probiotics.

## 1 Introduction

Respiratory and gastrointestinal (GI) viral infections represent a significant cause of morbidity and mortality worldwide (Gambadauro et al., 2024). Recent studies have underscored the pivotal role of gut microbiota in regulating host immune function and enhancing defense mechanisms against these infections (Anandakumar et al., 2024). Specifically, a healthy gut microbiome contributes to the maintenance of an effective immune response, enabling the host to combat viral infections more efficiently. This robust immune system not only targets the viral pathogens but also plays a crucial role in reducing the risk of secondary bacterial infections in affected patients (Oliva and Terrier, 2021).

The gut microbiota contains billions of microorganisms and at least 1,000 different bacterial species from 12 phyla. The microbiome consists of over 5,000,000 genes, 150–500 times larger than the human genome. Under normal physiological conditions, the complex interactions between the gut microbiota and the host immune system have co-evolved to maintain a symbiotic relationship, jointly preserving host homeostasis through host regulatory pathways and maintaining tolerance to innocuous antigens (Cheng et al., 2019; The Human Microbiome Project Consortium, 2012).

Numerous studies have demonstrated that gut microbiota play a significant role in regulating viral replication and shaping host antiviral immune responses, both locally and systemically. However, the precise mechanisms underlying these interactions—such as the roles of microbial metabolites, immune cell crosstalk, and signaling pathways—remain incompletely characterized. The gut is a critical site for controlling the development of immune cells in the gut and beyond. The gut microbiota plays a crucial role in maintaining immune homeostasis through the production of metabolites and antimicrobial peptides, which confer resistance against diverse pathogens, thereby modulating systemic immunity (Prasad et al., 2024). Additionally, it provides essential materials for fecal microbiota transplantation (FMT) and has been proven effective in treating patients with recurrent Clostridium difficile infections and other conditions (Fan and Pedersen, 2021). In line with this, FMT has shown promise in enhancing antiviral responses. For example, FMT has been shown to induce the clearance of HBeAg in HBeAg-positive patients undergoing long-term antiviral therapy, effectively suppressing viral replication (Chauhan et al., 2021). These findings underscore the potential of FMT as a therapeutic strategy to enhance antiviral efficacy. Furthermore, extensive research has elucidated the complex interactions between microbiota and T cells, demonstrating the protective effects of microbiota against lung infections (Byun et al., 2024; Jeyanathan et al., 2022). Thus, the gut microbiota may significantly influence the host immune response during viral infections.

To our knowledge, few studies have evaluated the dynamics and underlying mechanisms of the gut microbiota in patients with viral infections. Some studies have shown that lipopolysaccharides from symbiotic bacteria interact with influenza virus particles and alter their morphology, thereby reducing the stability of the influenza A virus (Resiliac et al., 2023). *Segmented filamentous bacteria* (SFB) induce adaptive immune responses in mice, such as cell differentiation and IgA production of gut T helper type 17 (Th17) (Park et al., 2023). Methods to regulate the gut microbiota, such as probiotics, positively affect host antiviral therapy, reducing the severity of symptoms and shortening the course of the disease (Zhang et al., 2021).

Here, we provide an overview of the interaction between the gut microbiota and the immune system during host infection with different types of viruses. Finally, this review outlined the impact of symbiotic bacteria, including specific probiotics, on gut microbiota composition and their protective roles in host viral infections. The goal is to offer new theoretical insights and molecular strategies for controlling viral infections while exploring more effective and safer methods for microbial regulation and identifying potential targets.

## 2 Composition and function of gut microbiota

Recently, in contrast to traditional bacterial culture techniques, approaches such as 16S ribosomal RNA gene (rRNA) sequencing and shotgun metagenomic sequencing have significantly advanced our understanding of microbial community diversity. The gut microbiota encompasses a diverse ecosystem of microorganisms, including bacteria, fungi, viruses, and protists, which collectively contribute to host physiology and health. The bacteria in the human gut microbiota can be classified into phyla, classes, orders, families, genera, and species (MetaHIT Consortium et al., 2010; She et al., 2024). In a comprehensive review of scientific literature and culture collection databases, 2,171 prokaryotic species related to humans were listed, belonging to 12 different phyla, mainly concentrated in the *Proteobacteria, Firmicutes, Actinobacteria*, and *Bacteroidetes* phyla. This diversity highlights the immense microbial presence in the human body, with the number of bacterial in a standard adult male being approximately equal to the number of human cells (Sender et al., 2016).

The gut microbiota, in turn, interacts closely with the host physiology, and the intestinal mucus network plays a crucial bidirectional regulatory role in this interaction (Chen et al., 2024). This mucus primarily consists of the high molecular weight glycoprotein mucin MUC2 (mouse Muc2) and forms a natural hydrogel barrier. On one hand, this barrier prevents direct contact between microorganisms and epithelial cells, thereby mitigating the risk of microbial translocation and inflammation. On the other hand, it serves as a nutrient source for specific symbiotic bacteria. For instance, *Akkermansia muciniphila* degrades mucins to obtain carbon and nitrogen for growth and metabolism. Moreover, the metabolites produced by these bacteria during mucin degradation, including short-chain fatty acids (SCFAs), feedback to regulate mucus secretion, thereby helping to maintain the integrity of the mucus barrier (Ioannou et al., 2024). In addition to their role in mucus regulation, SCFAs, which are abundantly present in the colon, have been shown to play a crucial role in modulating the functions of immune cells, such as T cells and dendritic cells (DCs) (Belkaid and Hand, 2014).

Host diet, genetic and immune factors, and the environment influence the complex gut microbiota. For instance, SCFAs such as acetate, propionate, and butyrate, which are derived from the fermentation of dietary fibers, play a pivotal role in energy homeostasis (Han et al., 2023). Butyrate serves as a primary energy source for colonocytes, while acetate and propionate are transported to peripheral tissues, where they contribute to gluconeogenesis and lipid metabolism (Hays et al., 2024). These metabolites also act as signaling molecules, engaging G protein-coupled receptors (GPCRs) such as GPR41 and GPR43 to regulate appetite, insulin sensitivity, and energy expenditure, thereby linking microbial metabolism to host metabolic health (Zhang et al., 2023). Beyond metabolites, gut microbiota-derived secreted proteins and extracellular vesicles (EVs) are critical mediators of microbial communication and host-microbe interactions. Secreted proteins, such as bacteriocins and enzymes, enable microbes to compete for ecological niches by inhibiting the growth of competing species or degrading complex substrates (Kang et al., 2024).

Interestingly, even in healthy individuals, there are significant differences in the diversity and abundance of specific microbial taxa that serve as indicators of gut ecosystem health, while the metagenomes of metabolic pathways remain stable across individuals (The Human Microbiome Project Consortium, 2012). This stability highlights the role of functional redundancy in maintaining gut health, as different microbial taxa can perform overlapping metabolic functions. As a result, the conservation of these functional pathways ensures the continuity of essential processes, such as nutrient metabolism and immune modulation, even when there are variations in the composition of the microbial community (Jones, 2010).

Recent studies have shown that the interaction between the gut microbiota and the host immune metabolic system can affect the related functions of other organs, forming axes such as the gut-lung axis (Ngo et al., 2024), gut-brain axis (Mayer et al., 2022), gut-kidney axis (Lan et al., 2023), and gut-liver axis (Nie et al., 2024). It is intriguing that the gut-lung axis, a relatively novel and less-studied bidirectional connection, links gut microbiota with the respiratory immune system, underscoring the potential impact of gut health on respiratory conditions—particularly in light of recent findings related to COVID-19 (Daoust et al., 2021). The perturbation of the symbiotic equilibrium between the gut microbiota and the host has been implicated in the etiology of a spectrum of disorders, encompassing inflammatory bowel disease (IBD) (Shan et al., 2022), obesity (Van Hul and Cani, 2023), psychosocial conditions (Kronsten et al., 2022), and autoimmune diseases (Zhang et al., 2020). Symptoms of dysbiosis can vary from mild gastrointestinal discomfort to more severe chronic illnesses, highlighting the importance of maintaining a balanced gut microbiome for overall health. Beneficial microbes, such as *Lactobacillus* and *Bifidobacterium*, contribute to maintaining intestinal barrier integrity, preventing pathogen invasion, and producing metabolites like SCFAs, which have anti-inflammatory properties. These mechanisms underscore the pivotal role of symbiotic bacteria in promoting host health and preventing disease.

## 3 The mechanism of gut microbiota in antiviral immunity

### 3.1 Gut microbiota interactions with immune cells during viral infection

**Figure 1:**
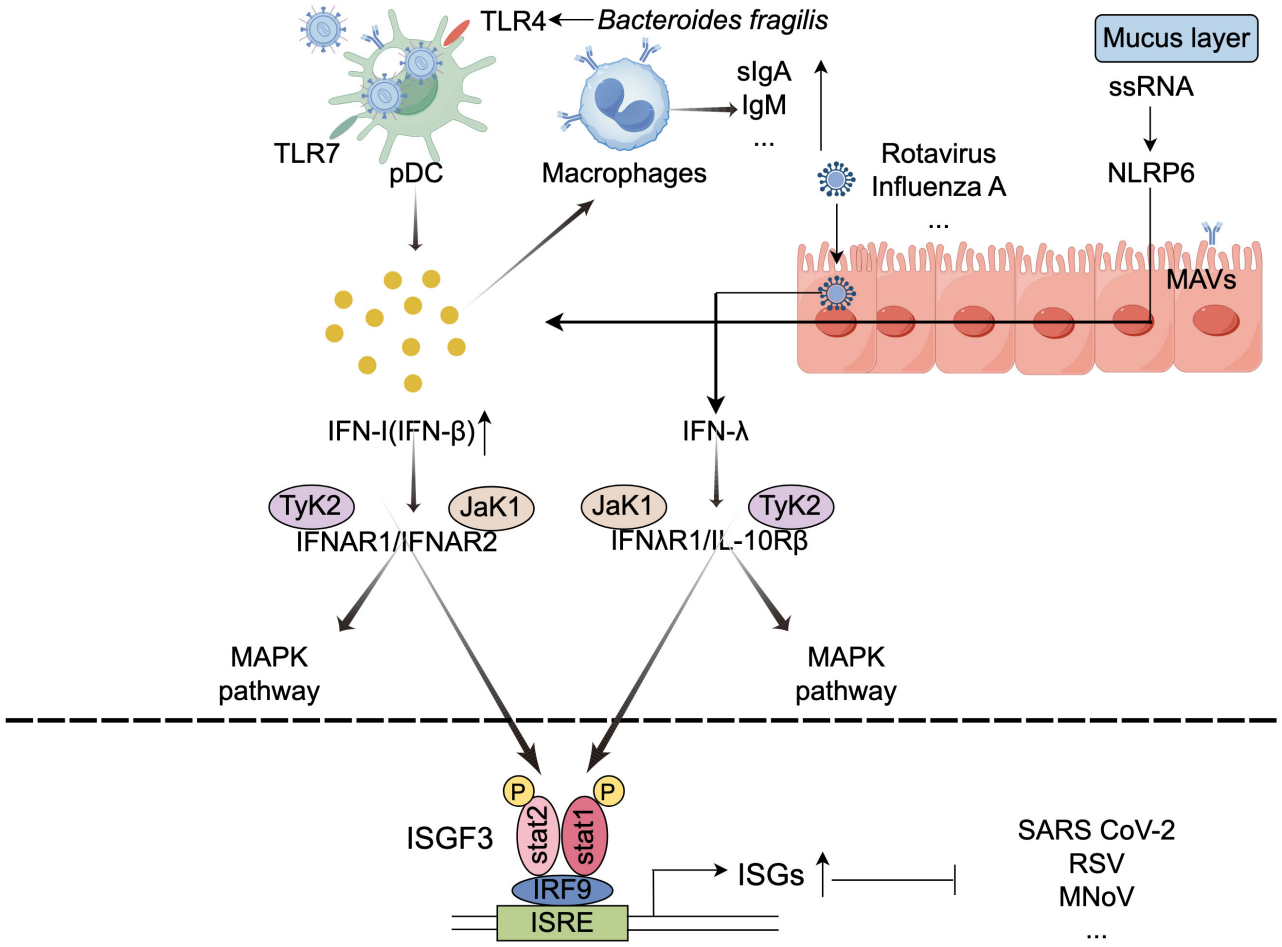
Mechanisms underlying the activation of immune cells by the intestinal microbiota (by Figdraw). The gut microbiota activates immune cells, such as macrophages and dendritic cells, by binding to pattern recognition receptors, which promotes the production of interferons and other immune factors. This stimulation of host cells leads to the transcription of ISGs that encode various antiviral proteins. TLRs, Toll-like receptors; ISGs, interferon-stimulated genes; ISGF3, ISG factor 3; IRFs, interferon regulatory factors; ISRE, IFN-stimulated response elements; ↓, decrease; ↑, increase.

The vast majority of bacterial cells colonize the lower GI tract, where they play a critical role in regulating both local and systemic immune responses (Stefan et al., 2020). Traditionally, the interaction between the symbiotic microbiota and the immune system was thought to primarily maintain tolerance to harmless antigens. However, emerging evidence highlights the gut microbiota's active role in antiviral immunity. When viruses invade the respiratory or gastrointestinal mucosa, the host employs multiple defense mechanisms, including the mucus layer, innate immunity, and adaptive immunity, to counteract infection (Harper et al., 2021). The gut microbiota acts as an immunological adjuvant, shaping immune system development and function. Immune cells are strategically localized at sites of bacterial colonization to maintain tissue homeostasis and coordinate immune responses. Furthermore, the gut microbiota modulates cytokine production, influencing immune activity not only locally but also at distal non-GI sites, thereby providing systemic protection against viral infections (Belkaid and Hand, 2014). Figure 1 illustrates the key mechanisms by which the intestinal microbiota activates immune cells.

Interferons (IFNs) are cytokines secreted by host cells in response to infections, and they specifically bind to receptors for viral nucleic acids (García et al., 2024). There are three classes of IFNs: type I, II, and III. Type I IFNs are produced by a wide range of cells, including immune and epithelial cells, while type II IFNs are primarily expressed by immune cells, and type III IFNs are predominantly produced by epithelial cells (Doldan et al., 2022). Upon infection, IFNs are produced and secreted through the activation of pattern recognition receptors (PRRs). The released IFNs can bind to their respective receptors in an autocrine (the infected cell itself) or paracrine (neighboring cells) manner (Mihaescu et al., 2023). When secreted IFNs bind to their homologous receptors, they activate transcription factors, such as signal transducer and activator of transcription (STAT) and interferon regulatory factors (IRFs), which in turn initiate the transcription of interferon-stimulated genes (ISGs) that encode various antiviral proteins (Zhou et al., 2020).

Type III IFNs have a more prominent protective effect on mucosal sites, such as the intestinal tract, airway, and hepatocytes (Wright and Nice, 2024). Although both type I and type III IFNs can activate mitogen-activated protein kinases (MAPKs), it is interesting to note that the antiviral activity induced by type III IFNs relies exclusively on the MAPK signaling pathway (Zhou et al., 2020). For example, the symbiotic bacterium B.coccoides activates IFN-I and ISG responses in mononuclear phagocytes through an IFNAR and STAT1-mediated signaling pathway, thereby inhibiting systemic enterovirus infection (Yang X.-L. et al., 2021). Similarly, C. scindens can activate type III IFNs by promoting the biotransformation of bile acids, which in turn inhibits murine norovirus (MNV) infection in the proximal small intestine of mice. Additionally, the absence of microbial communities leads to a severe attenuation of type I IFN and ISG responses in peripheral tissues and the brain, unrestricted viral replication, and an increase in the incidence and mortality of neurological disorders (Grau et al., 2019).

Recent studies have demonstrated that the gut microbiota interacts with antigen-presenting cells (APCs) to modulate antiviral immune responses. APCs, which are crucial for maintaining innate immunity, primarily include DCs, macrophages, and B cells. These cells bridge innate and adaptive immune responses, and their interaction is essential for the effective activation of naïve T cells (Gaudino and Kumar, 2019). Macrophages rely on signals from symbiotic bacteria to maintain a steady state of IFN response. For instance, depletion of gut microbiota in mice results in a deficiency of the macrophage antiviral response and affects the activation threshold of the interferon signaling pathway (Abt et al., 2012).

DCs are divided into three subgroups: plasmacytoid dendritic cells (pDCs), Type 1 conventional dendritic cells (cDC1), and Type 2 conventional dendritic cells (cDC2). pDCs are considered the major source of IFN-β under homeostatic conditions, mice lacking receptors for type I IFN or its signaling molecule STAT1 become extremely susceptible to viral infections (Ingle et al., 2018). cDCs rely on type I IFN signaling for functional maturation and migration (Marongiu et al., 2021). Intestinal DCs play a crucial role in the mucosal immune compartment, ensuring local induction of symbiotic bacterial immune responses without disrupting systemic immune responses.

Microbial-associated molecular patterns (MAMPs) (including gut microbiota composition, metabolites, and immunogenic components) are pattern recognition receptors (PRRs) involved in the expression of APCs in antiviral immune responses. According to their receptor types, PRRs can be categorized into constitutive and inducible expressions (Ahmadi Badi et al., 2021). Extracellular receptors, such as membrane-bound Toll-like receptors (TLRs), recognize bacterial flagellin and single-stranded viral RNA, activating the expression of interferons (IFNs). Research has shown that fragile Bacteroides glycosides induce IFNβ expression in colonic lamina propria DCs through the TLR4 signaling pathway, thereby enhancing resistance to viral infections. Animal experiments have confirmed the constitutive production of type I IFNs and the activation of interferon-stimulated genes (ISGs) (Stefan et al., 2020; Zheng et al., 2020). TLR4 is a key player in the immune response against COVID-19 infection (Baradaran Ghavami et al., 2021). Following respiratory influenza virus infection, gut microbiota regulates the Toll-like receptor 7 (TLR7) signaling pathway and airway mucosal immunity. The use of TLR7 ligands can rescue immune damage in antibiotic-treated mice (Wu et al., 2013). TLR7 also plays a crucial role in recognizing other viruses, including HIV and vesicular stomatitis virus (Zheng et al., 2023). Additionally, through TLR4 and MYD88-dependent signaling pathways, gut microbiota regulate granulocyte phagocytosis in mice (He et al., 2017).

Nucleotide oligomerization domain (NOD)-like receptors (NLRs) are central to the immune response against various microorganisms, responding to environmental insults and cellular danger signals. NOD2 recognizes single-stranded RNA (ssRNA) viruses and induces type I IFNs through mitochondrial antiviral signaling proteins (MAVS). Wang et al. found that Nlrp6 plays an important role in controlling intestinal viral infections in intestinal epithelial cells. It mainly binds to viral RNA through the RNA helicase Dhx15 and interacts with mitochondrial antiviral signaling proteins to induce type I/III interferons (IFNs) and ISGs (Wang et al., 2015).

The interaction between antigen-presenting cells (APCs) and T cells is essential for linking innate and adaptive immune responses (Gaudino and Kumar, 2019). DCs in the lamina propria capture viral particles, migrate to mesenteric lymph nodes, and present processed viral antigens to virus-specific T cells via major histocompatibility complex (MHC) class I and II molecules (Lockhart et al., 2022). This process is critical for the activation and differentiation of T cells, most of which are found in resident tissues such as the skin and gastrointestinal tract. The gut microbiota plays a pivotal role in shaping T-cell responses, particularly through the regulation of T-helper 17 (Th17) and regulatory T (Treg) cell differentiation. For example, colonization of germ-free mice with *Bacteroides fragilis* producing PSA induces CD4+ T-cell expansion and cytokine production, whereas Bacteroides fragilis lacking PSA does not elicit such effects (Mazmanian et al., 2005). Similarly, colonization with *Clostridium* species promotes the expansion of Foxp3 (+) T (Reg) and enhances interleukin-10 production in the colon, contributing to immune tolerance and homeostasis (Sano et al., 2021).

SFB are another key modulator of T-cell function. SFB colonization directly regulates the secretion of antimicrobial peptides and drives IL-17 production by Th17 cells, which are crucial for mucosal immunity. From birth, SFB colonization supports the functional maturation of intestinal T cells, influencing both innate and adaptive immune responses (Hathaway-Schrader et al., 2022). Additionally, *Lactobacillus* participates in tryptophan metabolism, producing aryl hydrocarbon receptor (AhR) ligands that promote IL-22 expression and further influence Th17 cell differentiation and amplification (Sun et al., 2020). SFB also plays a protective role against viral infections by stimulating the formation of isolated lymphoid tissues and tertiary lymphoid structures in the intestine, which facilitate T-cell-independent IgA production (Lécuyer et al., 2014). This mechanism enhances host defense against viruses such as influenza A virus, respiratory syncytial virus, and severe acute respiratory syndrome coronavirus infections, as evidenced by reduced viral loads and dampened pro-inflammatory gene expression in SFB-colonized mice (Ngo et al., 2024). In contrast, Candida albicans-induced Th17 cell activation has been shown to impair immunity against influenza A virus, highlighting the context-dependent roles of Th17 cells in antiviral immunity (Bacher et al., 2019). Furthermore, Bifidobacterium adolescentis promotes Th17 cell accumulation in the intestine through a transcriptional program distinct from that of SFB, underscoring the diverse strategies by which gut microbiota modulate T-cell responses. These findings collectively demonstrate the profound influence of the gut microbiota on T-cell differentiation and function, which in turn shapes antiviral immunity (Tan et al., 2016). Under the influence of pro-inflammatory and regulatory signals, the gut microbiota interacts closely with the mucosal immune system (Matson et al., 2021). In the human body, approximately 60% of CD4^+^ T lymphocytes are found in gut-associated lymphoid tissue. The intestinal mucosal immune system is one of the major targets of Human immunodeficiency virus (HIV) attack, and changes in the gut microbiota may affect the recovery of immune function. The imbalance of the gut microbiota in HIV-infected individuals is mainly manifested by changes in microbial diversity, a decrease in commensal probiotics, and an increase in potentially pathogenic bacteria. In addition, HIV-mediated CD4+T17 cell depletion and increased cytokine expression can lead to persistent immune cell activation and high circulating levels of inflammatory factors (Geng et al., 2020). These findings collectively demonstrate the profound influence of the gut microbiota on T-cell differentiation and function, which in turn shapes antiviral immunity.

The gut microbiota can regulate the immune response in distant mucosal areas of the body and have a significant impact on the mortality rate of respiratory viral infections (Nagata et al., 2023). A key mechanism underlying this regulation is the gut-lung axis, through which gut-derived signals modulate lung immunity. Group 2 innate lymphoid cells (ILC2s), which reside in tissues, are central to this cross-talk. Respiratory ILC2s not only mediate antiparasitic defenses but also contribute to antiviral immunity. Notably, gut-derived ILC2s can migrate into the bloodstream and influence the activation of lung-resident ILC2s, thereby enhancing local immune responses (Pu et al., 2021; Ricardo-Gonzalez et al., 2020). In addition to ILC2s, symbiotic bacteria regulate the adaptive immune response to respiratory viruses. Following influenza virus infection, the gut microbiota influences the production of virus-specific CD4+ and CD8+ T cells, as well as antibody responses. For instance, neomycin-sensitive bacteria have been shown to enhance lung immunity by maintaining stable expression of pro-IL-1β and pro-IL-18, which are critical for initiating inflammatory and antiviral responses (Ichinohe et al., 2011).

However, the precise bacterial components or signaling pathways mediating some microbiota-immune interactions remain unidentified. For instance, while certain gut bacteria have been shown to enhance antiviral immunity, the exact molecular mechanisms underlying these effects are not fully understood. In such cases, future research employing metagenomic and metabolomic analyses could help identify novel mediators and clarify their roles in immune regulation. By addressing these gaps, we can better leverage the gut microbiota's potential in antiviral therapies.

### 3.2 Production of immune-related molecules

**Figure 2:**
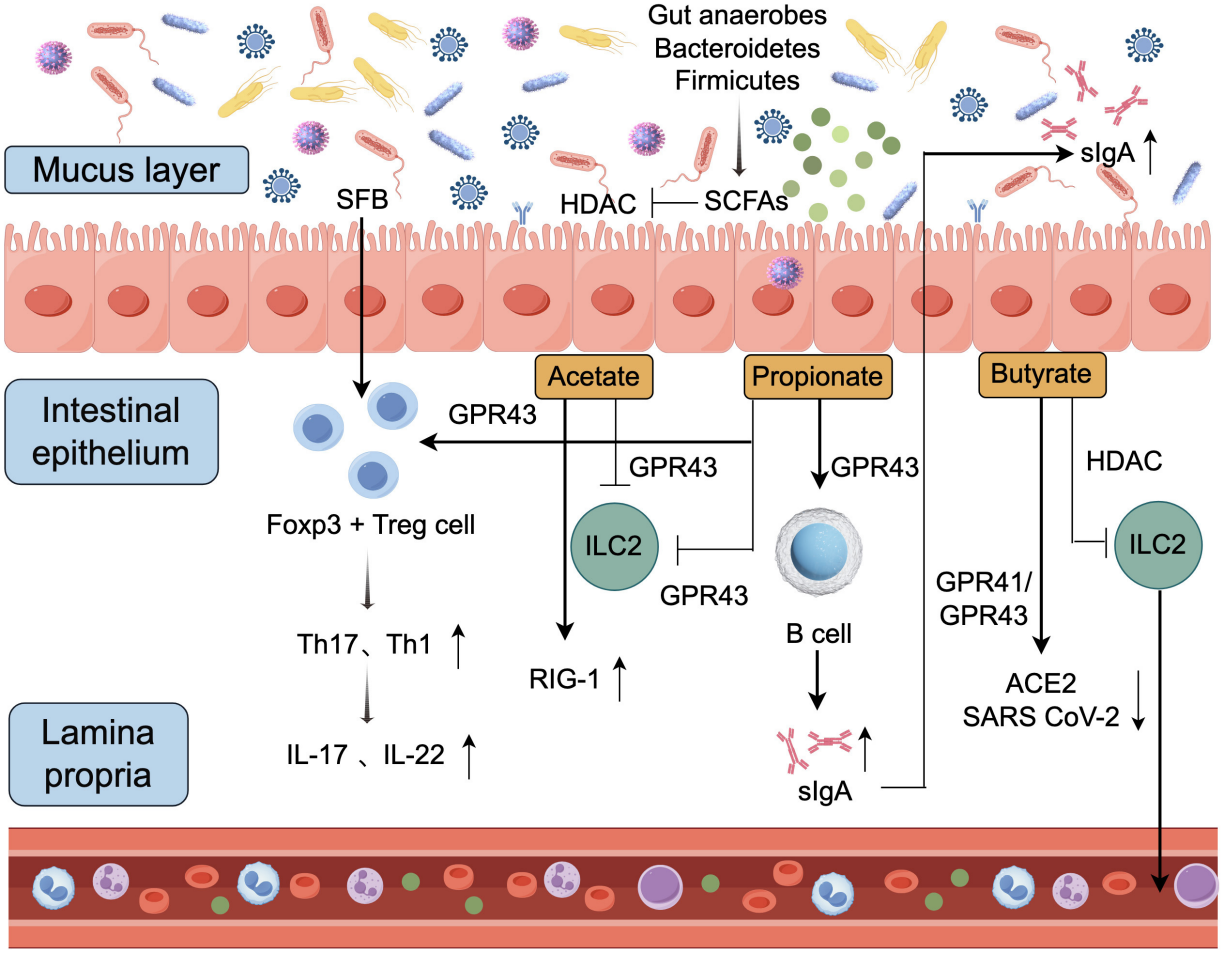
Mechanisms of SCFA-mediated antiviral activity (by Figdraw). Gut commensal bacteria produce SCFAs when metabolizing indigestible carbohydrates. These SCFAs can activate type I interferon expression through a GPR43-dependent pathway or enhance virus recognition via the RIG-1 signaling pathway, while also promoting the production of specific antibodies by immune cells. Tregs, T regulatory cells; SCFAs, short-chain fatty acids; HDACs, histone deacetylases; GPR41, G-protein coupled receptor 41; GPR43, G-protein coupled receptor 43; ILC2, type 2 innate lymphoid cells; IL, interleukin; ↓, decrease; ↑, increase.

The human gut microbiota is involved in the metabolism of carbohydrates that are difficult to digest, metabolites produced during this process, such as SCFAs, play a key role in mucosal immune responses. Notably, SCFAs mainly include acetate (from most gut anaerobes), propionate (form Bacteroidetes), and butyrate (form Firmicutes) which exert pro-viral or antiviral effects depending on the type of virus (Alwin and Karst, 2021). In addition to SCFAs, the gut microbiota regulates host immune responses through a variety of other metabolites. For instance, microbial-secreted proteins such as Amuc_1100 can enhance intestinal barrier function via the TLR2 signaling pathway (Plovier et al., 2017). Bile acid metabolites, such as secondary bile acids, modulate regulatory T cell differentiation through FXR and TGR5 receptors (Hang et al., 2019). Furthermore, bacterial cell wall components like peptidoglycan fragments can activate innate immune responses via NOD-like receptors (Clarke et al., 2010). These metabolites, together with SCFAs, form a complex network of microbe-host interactions. Figure 2 illustrates the antiviral mechanisms mediated by SCFAs in the context of gut microbiota.

The SCFAs are known to modify various cellular processes, acting mainly on various immune cells such as dendritic cells, macrophages, neutrophils, and regulatory T cells, and participating in local and distant immune regulation through multiple mechanisms (Jaye et al., 2022; Rosshart et al., 2019). By binding to specific G protein-coupled receptors (GPCRs, such as GPR41/FFAR3 and GPR43/FFAR2), short-chain fatty acids activate their anti-inflammatory effects (Yang et al., 2020). They can also be used as histone deacetylases (HDACs) inhibitors. SCFAs interact closely with other microbial metabolites. For example, bile acid metabolites can influence the expression of SCFA receptors (Parada Venegas et al., 2019), while bacterial-secreted proteins may regulate SCFA production (Louis and Flint, 2017). This synergistic interplay plays a crucial role in maintaining gut homeostasis and modulating systemic immune responses. In addition, SCFAs are not only involved in the metabolic processes of immune cells but can also influence the differentiation of helper T cells or promote the rapid response of CD8+ T memory cells through the mTOR signaling pathway after being converted to acetyl-CoA and integration into the Krebs cycle (Jing et al., 2023).

Among the most abundant SCFAs in the colon, acetate is predominantly produced by acetogenic bacteria such as Blautia, accounting for nearly one-third of total acetate (Verstraeten et al., 2022). An *in vitro* study showed that acetate regulates mouse respiratory syncytial virus infection through the RIG-1 signaling pathway, RIG-1 knockdown of A549 cells lacks a protective effect against respiratory syncytial virus (Antunes et al., 2022). Research has shown that high-fiber dietary sources of acetate promote the production of type I IFN and the expression of ISG in lung epithelial cells through GPR43, thereby reducing virus-induced cytotoxicity and promoting antiviral effects (Antunes et al., 2019). SCFA acetate enhances the intestinal IgA response through GPR43, with the underlying mechanism involving acetate-induced expression of Aldh1a2 in DCs. This process converts vitamin A into its metabolite, retinoic acid (RA), which in turn promotes IgA production by B cells. This discovery could uncover a novel mechanism by which the microbiota regulates intestinal homeostasis. Additionally, acetate activates the mTOR pathway in DCs, and inhibiting mTOR can suppress acetate-induced IgA production by B cells (Sepahi et al., 2021).

Treatment with SCFA propionate enhanced the phagocytic ability of mouse DCs but inhibited activating Th2 cell function (Trompette et al., 2014). This resulted in reduced expression of CD40, programmed death ligand 2 (PD-L2), and CD86. Numerous reports indicate that SCFAs can mediate Treg cell differentiation. In addition to acetate, propionate can promote local aggregation of Tregs by activating GRP43 on the surface of target cells and regulating gene expression by inhibiting HDACs (Smith et al., 2013). However, SCFAs above physiological levels induce dysregulation of Th1 and Th17 cell responses in mice and also have the potential to induce inflammatory responses (Campos-Perez and Martinez-Lopez, 2021). Previous research has indicated that the proportion of Firmicutes and Bacteroidetes in the intestines and lungs of mice fed a high-fiber diet tends to increase (Sun et al., 2023). Furthermore, B lymphocytes utilize acetyl-CoA produced by SCFAs to engage in cellular metabolism, thereby supporting the production of pathogen-specific antibodies (Antunes et al., 2023; Kim, 2021).

Butyrate is the main energy source for colon cells and also participates in the processes of fat formation and gluconeogenesis in peripheral tissues (Zhao et al., 2021). Experiments have shown that butyrate supports the health of distant organs, and a negative correlation has been identified between butyrate-producing gut bacteria (BPG) and the risk of lower respiratory tract viral infections in kidney transplant recipients (Lee et al., 2019). Recently, studies have indicated that dietary fiber plays a key role in balancing both innate and adaptive immunity. Feeding mice with butyrate reduces the production of the chemokine CXCL1 in the airways, thereby decreasing neutrophil infiltration and limiting the progression of influenza virus in mice (Zhang H. et al., 2024). The primary mechanism involves butyrate-mediated activation of FFAR3 on bone marrow cells, which promotes the differentiation of monocytes into anti-inflammatory macrophages in the lungs. These macrophages produce lower levels of CXCL1, thereby reducing inflammation compared to their inflammatory counterparts. In addition, after Epstein-Barr Virus (EBV) infection, propionic acid and butyric acid were found to activate the EBV lysis cycle. They have also been found to be universal inhibitors of class I and class II histone deacetylases (HDACs) in various cell types. They can also limit the proliferation of ILC2s in the lung (Eladwy et al., 2023; Liu et al., 2023). Butyrate salts have HDAC inhibitory activity both *in vitro* and *in vivo* and can remove the acetyl group on histone ε-N-acetyllysine, allowing histones to wrap more tightly around DNA (Choudhary et al., 2009).

However, in contrast to previous reports that butyrate regulates the type II IFN response, *in vitro* studies have shown that the short-chain fatty acid butyrate also reprograms the type I IFN response by regulating the induction of specific ISGs, leading to increased viral replication. This result may also be related to the HDAC inhibitor properties of butyrate, the use of immortalized cell lines, and the different viruses tested (Chemudupati et al., 2020). Butyrate also inhibits the proliferation of helper T cell subsets, including Th1, Th17, and Th22, while reducing the production of cytokines by these cells (Kibbie et al., 2021). Additionally, butyrate promotes extrathymic generation of Treg cells in an intronic enhancer CNS1 (conserved non-coding sequence 1) and prevents the progression of systemic inflammation (Hu et al., 2022). RIG-I-like receptors, RLRs, such as retinoic acid-inducible gene I(RIG-I) and MDA5, are a type of cytoplasmic PRRs that specifically recognize viral RNA, promote secretion of type 1 IFNs, and participate in viral recognition (Sharma et al., 2021). Recent studies have found that exogenous butyrate supplementation can induce RIG-I expression and promote rhinovirus clearance (Antunes et al., 2023). Similarly, research on murine norovirus (MNoV) models has shown that during the process of virus replication in host cells, innate immune receptors (such as TLR RIG-1, MDA) can sense viral molecules and further produce type I interferon response to eliminate infection (Lucero et al., 2021). GPCRs have specific binding abilities for different SCFAs. Butyric acid tends to bind more to GPR41 receptors, while GPR43 receptors have a higher affinity for acetic acid and propionic acid (Wang et al., 2024). Male mice treated with SCFAs exhibited reduced angiotensin-converting enzyme 2 (ACE2) expression and viral load, alongside improved adaptive immunity, through the activation of GPR41 and GPR43 receptors (Brown et al., 2022).

In summary, the gut microbiota shapes host immunity through a diverse array of metabolites, including SCFAs, secreted proteins, bile acid metabolites, and cell wall components. These metabolites act through distinct molecular mechanisms and signaling pathways, working synergistically to regulate both local and systemic immune responses. Future research will focus on using microbial-deficient mouse models to study the effects of specific metabolites on host immune responses.

### 3.3 Modulation of intestinal barrier function

**Figure 3:**
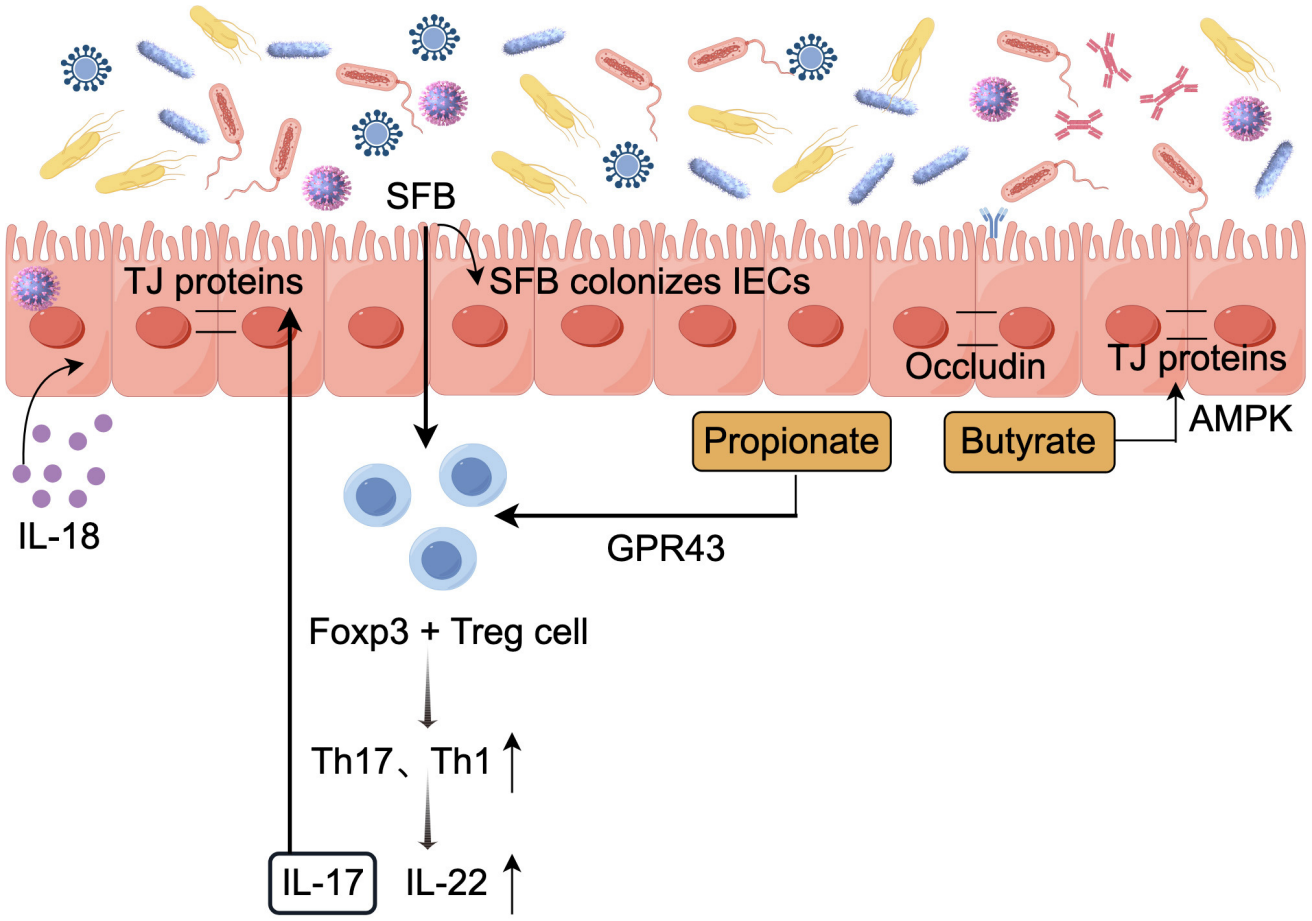
Role of the gut microbiota in maintaining mucosal barrier integrity (by Figdraw). The gut microbiota helps maintain the integrity of the mucosal barrier, thereby reducing the risk of infection. TJ, Tight junction; ↓, decrease; ↑, increase.

The intestinal barrier is a complex and dynamic system composed of physical and chemical structures, appropriate permeability characteristics of intestinal epithelial cells (IECs) are crucial for their barrier function (de Vos et al., 2022). Gut microbiota is involved in regulating the integrity of the mucosal barrier, and the relationship between gut microbiota and barrier permeability has been extensively studied in various disease models (Rastogi and Singh, 2022). Several proposed mechanisms include (i) Modulating intracellular transcription and translation to activate host immune protection pathways; (ii) Colonization of the intestinal epithelium by commensal bacteria reduces the attachment of pathogenic bacteria; and (iii) Metabolites act on IECs, reducing virus adsorption and entry. Figure 3 depicts the role of the gut microbiota in preserving the integrity of the mucosal barrier.

The mucus layer, primarily composed of Mucin 2(MUC2) secreted by intestinal epithelial cells, serves as the first line of defense against viral infections. External recognition of intestinal epithelial cells triggers a signaling cascade that activates transcriptional programs, produces antimicrobial effector proteins and secreted factors, and alerts both innate and adaptive immune cells (Segrist and Cherry, 2020). In the intestine of mice without specific pathogens, the bacterial microbiota activates a balanced ISG signaling pathway. This balanced expression of ISG is mainly restricted to IECs and depends on the IFN-λ receptor (IFNLR1) expressed by these cells. Meanwhile, this expression is closely related to the production of IFN-λ by white blood cells. Specifically, the IFN-λ response is highly localized in intestinal tissues, and analysis of multiple scRNA-seq datasets from mouse and human intestinal IECs describes that steady-state ISGs are mainly expressed in the mature epithelium at the tips of the villi (Van Winkle et al., 2022). Furthermore, *C Rodentium* and enterohemorrhagic Escherichia coli O157(EHEC O157) adhere to IECs to induce host-specific TH17 cell responses. Microorganisms adhering to SFBs and other ECs can be engulfed by intestinal epithelial cells and further processed by apoptotic ECs and loaded onto MHC-II molecules to exert their effects (Atarashi et al., 2015).

During chronic lymphocytic choriomeningitis virus (LCMV) infection in mice, the virus replicates in hematopoietic and mesenchymal cells in the small intestine. The gut microbiota causes dysregulation of epithelial cell tight junctions through type I interferon signaling, enhances the CD8+ T cell response, and continuously increases the paracellular permeability of the small intestine to low molecular weight (4KD) molecules. This is also associated with increased incidence and mortality following secondary chemical or microbial intestinal injury (Labarta-Bajo et al., 2020). Rotavirus (RV) primarily infects mature intestinal epithelial cells, leading to atrophy of intestinal villi, increased epithelial cell turnover, and apoptosis (Amimo et al., 2021). Evidence suggests that Lactobacillus casei and Bifidobacterium adolescentis metabolites maintain barrier permeability by reducing the expression of rotavirus enterotoxin NSP4 and Ca2+ release levels (Olaya Galán et al., 2016).

The intestinal epithelial barrier and epithelial renewal processes are regulated by pattern recognition receptor signaling pathways, emphasizing the specific modulation by TLRs. The gut microbiota activates TLR signaling in the small intestinal epithelium via epithelial nerve ciliary protein-1 (NRP1), thereby regulating the Hedgehog (Hh) pathway and modulating the intestinal barrier. Notably, in germ-free mice, elevated levels of epithelial NRP1 are associated with a strengthened intestinal barrier. In contrast, specific loss of epithelial NRP1 results in microbiota-dependent inhibition of Hh signaling, which is linked to a weakened intestinal epithelial barrier (Pontarollo et al., 2023). In antibiotic-treated mice, it was found that gut microbiota plays a crucial role in regulating TLR7 signaling, which helps alleviate damage to the common mucosal immune system (MIS) caused by the antibiotic treatment (Zhang Y. et al., 2024).

Tight junction (TJ) proteins include transmembrane proteins such as tight junction proteins and occludins, as well as cytoplasmic scaffold proteins from the ZO family, which serve both connecting and blocking functions, play a crucial role in regulating paracellular permeability and barrier function (Gou et al., 2022). Symbiotic bacteria have been shown to inhibit pathogen colonization by competing for specific metabolites, known as colonization resistance, colonization by symbiotic bacteria can regulate the expression of molecules related to tight structures. Recent studies have found that after depleting the gut microbiota through antibiotic treatment, the expression of tight junction protein ZO-1 and barrier function-related molecules, including Axin2, Claudin-2, and Claudin-3, is downregulated (Guo et al., 2021). Moreover, the *Lactobacillus plantarum* strain ZLP001 significantly inhibits the increase in intestinal permeability caused by infection. Its mechanism may involve stabilizing the tight junction structure of intestinal epithelial cells by preserving the abundance of tight junction proteins (Wang et al., 2018). Meanwhile, SFB colonization in the ileum induces significant changes in host gene expression, promoting the proliferation and migration of intestinal epithelial cells, and induces tight junction proteins, which helps reduce rotavirus replication levels (Ngo et al., 2023).

SCFAs produced by the gut microbiota act as nutrient supplements that modulate intestinal barrier function. SCFAs reduce the growth and attachment of pathogenic microorganisms and enhance epithelial integrity by reducing the intestinal pH (Yang M. et al., 2021). Elhaseen et al. (2013) study demonstrated that butyrate primarily enhances the protective effect on the intestinal barrier function of Caco cell monolayers through AMP-activated protein kinase (AMPK) activation. For example, Butyrate can upregulate MUC2 expression in these cells, enhancing mucosal protection (Jung et al., 2022). Furthermore, butyrate regulates the assembly of tight junctions through AMPK activation, thereby enhancing the stability of the intestinal barrier *in vitro* in colon epithelial cells. However, studies have shown that *in vitro* models indicate a positive correlation between the concentration of butyrate and its impact on intestinal barrier function, Physiological concentrations of butyrate (≤ 2 mM) promote intestinal barrier function (Peng et al., 2009), whereas higher concentrations (5 or 8 mM) may disrupt it by inducing apoptosis in cells (Huang et al., 2014). IL-18 plays a vital role in repairing and maintaining epithelial cell integrity, while SCFAs enhance IL-18 production by activating FFAR2 and GPR109a receptors in IECs (Macia et al., 2015).

As research into host-microbiome interactions advances, there is increasing focus on how gut microbiota affect fundamental cellular processes in intestinal epithelial cells, including protein turnover (Arike et al., 2020), and the circadian rhythms of food intake and processing (Brooks et al., 2021). Additionally, this understanding can be applied to address potential causes of intestinal barrier dysfunction, such as inflammatory bowel disease and cancer.

## 4 Antiviral potential of interventions altering the microbiota

Over the past decade, research has gradually revealed the role of the gut microbiota in shaping the host's antiviral immune response. Probiotics are defined as “live microorganisms which when administered in adequate amounts confer a health benefit on the host” (Hill et al., 2014), mainly including *Lactobacillus, Bifidobacterium*, Lactococcus, *Streptococcus, Enterococcus, Bacillus, Clostridium*, and *Saccharomyces*. While probiotics exert various beneficial effects on the host, it is important to note that most probiotic strains are transient colonizers in the gastrointestinal tract. Studies have demonstrated that the majority of ingested probiotics are eliminated from the gut within days to weeks after cessation of supplementation (Shen et al., 2024). Their transient presence can still benefit the host through multiple mechanisms: (1) inhibiting pathogens via competition and antimicrobial production; (2) blocking pathogen adhesion; (3) Probiotic-derived metabolites, including SCFAs, tryptophan catabolites, and CLAs, mediate indirect immunomodulation through multiple signaling pathways; and (4) modulating immune responses through cytokine regulation (Bhutta et al., 2024; Rau et al., 2024). Currently, many commercial probiotics consist of specific strains of Lactobacillus and Bifidobacterium (Hill et al., 2014). A growing body of research literature shows that animals receiving broad-spectrum antibiotic combination therapy and germ-free mice (GF mice) are impaired in both innate and acquired immune responses after infection with various viral pathogens, including norovirus, LCMV, influenza virus, vesicular stomatitis virus (VSV), and others (Santiso-Bellón et al., 2022). Regulating symbiotic bacterial communities holds significant therapeutic potential. Different types and strains of probiotics influence the outcome of host diseases in various ways, primarily by regulating immune responses, enhancing intestinal barrier function, and competitively inhibiting pathogenic microorganisms.

Direct immunomodulation by probiotics occurs through MAMPs engaging with host pattern recognition receptors. For instance, oral administration of *Lactobacillus plantarum* 0111 regulates the host immune system and upregulates IFN in the intestine and lungs of H9N2-infected mice—β transcription levels and related ISGs, while increasing CD3^+^CD4^+^TNF-α ^+^ T lymphocyte and CD3^+^CD8^+^TNF- α ^+^ T lymphocyte percentage in the spleen and reduces the severity of viral infection (Xing et al., 2022). Notably, heat-inactivated plant lactobacilli provide long-lasting protection against the fatal consequences of lung virus infection, with a 60% survival rate sustained for 13 weeks post-inoculation. Additionally, lactic acid bacteria intervention extended the survival of MyD88 gene-deleted (MyD88 (-/-)) mice and safeguarded them from the fatal sequelae of pneumonia virus infection, suggesting that the protective mechanism may operate independently of TLR (Gabryszewski et al., 2011). Also, Gutierrez-Merino et al. (2020) reported that symbiotic lactic acid bacteria (LAB) recognize and trigger type I interferon production via stimulator of interferon genes (STING) and mitochondrial antiviral signaling protein (MAVS) in human macrophages. In animal model research, *Lactobacillus paracasei* was found to increase the recruitment of dendritic cells in mouse lung tissue after influenza A virus infection (Belkacem et al., 2017). After 6 weeks of taking Lactobacillus paracasei (L. *casei* 431^®^), healthy adults showed an improved immune response following the seasonal influenza vaccine, with significant increases in vaccine-specific plasma IgG3, IgG1, and IgG levels (Rizzardini et al., 2012). The Lactobacillus paracasei (MI29) identified in the feces of healthy volunteers showed enhanced IFN-I signaling in vitro. Compared to the control group, mice treated with MI29 had a significant increase in the number of CD11c+PDCA-1+ plasma cell-like dendritic cells and Ly6Chi monocytes in their lungs (Kim et al., 2023). In a mouse model of lethal influenza virus infection, oral administration of *Lactobacillus pentosus* b240 prior to infection significantly extended survival. In cases of non-lethal infection, daily oral administration of b240 exhibited a substantial reduction in lung virus titers by the 7-day post-infection (Kobayashi et al., 2011). Influenza virus infection significantly increases TLR7 mRNA expression in lung immune cells. However, antibiotic-induced dysregulation reduces the expression of TLR7 signaling pathway genes. Probiotic intervention can restore the upregulation of TLR7 and other related genes, thus properly activating the inflammatory response (Gao et al., 2023).

Recent clinical studies have demonstrated that Bifidobacterium strains can elevate levels of IFN-γ, IL-2, tumor necrosis factor (TNF), serum IgA, and IgA-secreting cells during upper respiratory infections (Raheem et al., 2021). In a BALB/c mouse model infected with influenza A (H1N1) virus, prophylactic administration of bifidobacteria markedly boosted both humoral and cellular immunity, significantly lowered pulmonary IL-6 levels, and improved survival rates (Mahooti et al., 2019; Marrella et al., 2024). The role of Bifidobacterium longum in mitigating rotavirus infections by reducing viral infectivity is also well established. However, the effectiveness of probiotics is constrained by their survival and abundance in the gastrointestinal tract, with an intake of at least 10^∧^6 CFU/g required to confer significant health benefits. To enhance their survival and efficacy, researchers have explored combining probiotics with prebiotics. For instance, compared to untreated infected cells, the combination of Bifidobacterium *longum* and certain prebiotics improved the monolayer integrity of rotavirus-infected cells and enhanced the cellular antiviral response (Romero-Arguelles et al., 2023). Emerging evidence indicates that prebiotics may modulate viral infections through microbiota-mediated mechanisms. Notably, specific prebiotics such as galactooligosaccharides (GOS) have been shown to selectively enrich mucin-degrading bacteria like *Akkermansia* muciniphila and stimulate the production of immunomodulatory metabolites, including pentadecanoic acid (C15:0). These microbial changes collectively contribute to the alleviation of intestinal inflammation and restoration of epithelial barrier integrity (Wu et al., 2024). Similarly, fructooligosaccharides (FOS) influence host immunity by enriching Bifidobacterium, enhancing butyrate production, and modulating mucosal immune responses, thereby strengthening gut barrier function (Slavin, 2013). It is known that the host's defense against influenza A virus (IAV) involves the activation of the NLRP3 inflammasome. The activation of inflammasomes is a crucial innate immune mechanism that defends against pathogen invasion by secreting pro-inflammatory cytokines IL-1β and IL-18 and inducing cell pyroptosis (Coll and Schroder, 2024). A study found that the gut microbiota of Nlrp3-/- mice is enriched with a new bacterium, *Bifidobacterium pseudolongum* NjM1, which can protect wild-type mice from IAV infection during cohabitation. This suggests a potentially mutual regulatory relationship between the host's genetic factors and the gut microbiota (Niu et al., 2023).

Probiotic preparations can effectively enhance the mucosal immune response to influenza vaccination (Yakabe et al., 2021). P40 protein derived from the *Lactobacillus rhamnosus* GG (LGG) probiotic strain stimulates *April* gene expression in mouse intestinal epithelial cells and further stimulates B cells to produce IgA (Wang et al., 2017). The use of LGG is also significantly associated with a reduced risk of acute infectious diarrhea in infants and young children (Lukasik et al., 2022). In addition, strains of lactic acid bacteria have been found to enhance mucosal immunity, potentially helping to prevent the entry and replication of COVID-19 in respiratory and gastrointestinal tissues, while also possibly alleviating the excessive immune responses observed in severe COVID-19 cases (Taufer et al., 2024). While lactic acid bacteria show potential in enhancing mucosal immunity against COVID-19, their immunomodulatory effects, including potential anti-inflammatory properties mediated by unidentified soluble factors, require further mechanistic investigation (Decout et al., 2024).

Probiotics can limit the attachment and entry of pathogenic viruses by competing for binding sites on host cells. Research has shown that substances produced by certain strains of lactobacilli, such as bacteriocins and exopolysaccharides, can inhibit viral replication and protect the body from infection (Wang et al., 2022). Anwar et al. (2021) found through molecular dynamics studies of Lactobacillus plantarum metabolites that these substances bind to ACE2, RdRp, and RBD, effectively preventing the virus from entering host cells. Feeding *B. thermophilum* RBL67 before infection can inhibit the replication of RV in intestinal cells, thereby reducing the duration of diarrhea. This effect is achieved as *B. thermophilum* competes with RV for binding sites on host cells and enhances tight junctions between intestinal epithelial cells, which decreases the adhesion of intestinal cells to RV (Gagnon et al., 2016). Furthermore, phage therapy represents another promising approach to modulating viral infections. Recent studies have demonstrated that specific phages can target and reduce the load of pathogenic bacteria that may exacerbate viral infections. Phage-derived enzymes, such as endolysins, have shown potential to disrupt viral replication mechanisms (Schmelcher et al., 2012). Additionally, the interaction between phages and gut commensals can promote the stability and diversity of beneficial microbiota, further reinforcing gut barrier function and immune regulation. These findings provide a theoretical foundation and potential directions for the application of phage therapy in antiviral treatments (Gogokhia et al., 2019).

In addition to *Lactobacillus* and *Bifidobacterium*, probiotics that produce deaminotyrosine (DAT), such as *Lactobacillus pentosus* CCFM1227, initiate the type I interferon signal amplification, participate in improving lung tissue inflammation and activating platelets and TNF-a in the bloodstream, thereby exerting antiviral influenza effects (Wang et al., 2023). Zuo et al. found a correlation between the severity of COVID-19 hospitalization and the fecal microbiome. Their research indicated that patients with severe conditions have an enrichment of opportunistic pathogenic bacteria and a significant reduction in beneficial symbiotic bacteria. Interestingly, Faecalibacterium prausnitzii is among the bacteria most strongly negatively correlated with disease severity (Zuo et al., 2020). Based on current clinical trials, we speculate that probiotic administration might be more effective in alleviating virus-related illnesses that are more likely to alter the composition of the intestinal microbiota.

## 5 Conclusions and future perspectives

Recent studies have provided substantial evidence for the role of intestinal microorganisms in regulating host viral infectious diseases. This review summarizes some potential mechanisms that may help limit viral infections, focusing on the gut microbiota and its metabolites. These components may indirectly impact viral infections by altering cellular states and stimulating innate or adaptive immunity. Numerous preclinical and clinical studies have highlighted the specific immunomodulatory effects of these mechanisms. However, microbiota is a highly dynamic and complex microbial community, and the diversity of research methods has resulted in inconsistent findings in immunology, hematology, and clinical indicators. Therefore, it is essential to clearly describe the randomization process to minimize bias. Additionally, as our understanding of the microbiota's impact on viral infections evolves, the mechanisms by which gut microbiota influence health and disease require further investigation. More evidence is urgently needed to confirm their safety and effectiveness in clinical practice. Collectively, through research and analysis, we have explored the therapeutic potential of gut microbiota in viral infections and clarified the underlying mechanisms, thus laying the groundwork for the development and application of new therapies.

These innovative approaches—probiotics, prebiotics, phage therapy, and FMT are vital in treating both viral and non-viral diseases and maintaining homeostasis.
